# Alignment of a Trivalent Chromosome on the Metaphase Plate Is Associated with Differences in Microtubule Density at Each Kinetochore

**DOI:** 10.3390/ijms251910719

**Published:** 2024-10-05

**Authors:** Ashley B. Borseth, Hedyeh D. Kianersi, Paige Galloway, Grace Gercken, Emily L. Stowe, Marie Pizzorno, Leocadia V. Paliulis

**Affiliations:** Biology Department, Bucknell University, 1 Dent Dr., Lewisburg, PA 17837, USApag012@bucknell.edu (P.G.); estoweva@bucknell.edu (E.L.S.);

**Keywords:** chromosomes, meiosis, trivalent, chromosome alignment, metaphase

## Abstract

Chromosome alignment on the metaphase plate is a conserved phenomenon and is an essential function for correct chromosome segregation for many organisms. Organisms with naturally-occurring trivalent chromosomes provide a useful system for understanding how chromosome alignment is evolutionarily regulated, as they align on the spindle with one kinetochore facing one pole and two facing the opposite pole. We studied chromosome alignment in a praying mantid that has not been previously studied chromosomally, the giant shield mantis *Rhombodera megaera*. *R. megaera* has a chromosome number of 2*n* = 27 in males. Males have X_1_, X_2_, and Y chromosomes that combine to form a trivalent in meiosis I. Using live-cell imaging of spermatocytes in meiosis I, we document that sex trivalent Y chromosomes associate with one spindle pole and the two X chromosomes associate with the opposing spindle pole. Sex trivalents congress alongside autosomes, align with them on the metaphase I plate, and then the component chromosomes segregate alongside autosomes in anaphase I. Immunofluorescence imaging and quantification of brightness of kinetochore–microtubule bundles suggest that the X_1_ and X_2_ kinetochores are associated with fewer microtubules than the Y kinetochore, likely explaining the alignment of the sex trivalent at the spindle equator with autosomes. These observations in *R. megaera* support the evolutionary significance of the metaphase alignment of chromosomes and provide part of the explanation for how this alignment is achieved.

## 1. Introduction

Meiosis is a fundamental process for successful gamete formation and sexual reproduction. The formation of bivalents, followed by two rounds of division separating homologues in anaphase I and sister chromatids in anaphase II, ensures the correct reduction in chromosome number. Incorrect separation of partner chromosomes, or non-disjunction, leads to changes in chromosome number [1].

In general, bivalent formation is required for successful meiotic completion, as the two homologues that form the bivalent associate with microtubules coming from opposite poles, experience tension along their length, and are then separated from one another. Failures in the congression of chromosomes or in successful segregation can lead to aneuploidy. Aneuploidy is associated with health consequences in humans, such as cancer, congenital developmental delay, and miscarriage. Unusual combinations of chromosomes, however, occur routinely throughout the tree of life and segregate correctly in the organism that possesses the variation. One notable example is found in the many species of praying mantids that have a sex trivalent. Primary spermatocytes of trivalent-containing praying mantids have an X_1_ chromosome, an X_2_ chromosome, and a Y chromosome that combine to form the sex trivalent. Each of the three chromosomes possesses individual kinetochores where the two X chromosomes face one spindle pole and the one Y chromosome faces the opposing spindle pole [2,3,4,5,6,7,8,9]. Thus, the configuration of the sex trivalent leads to an intriguing question regarding how it can successfully navigate the meiotic program given seemingly unequal connections to spindle poles.

Sex trivalents have been documented across multiple kingdoms and are not uncommon in plants and arthropods [2,3,4,5,6,7,8,9,10,11,12]; however, the mechanisms that dictate chromosome alignment and segregation remain a mystery [13]. In most organisms in which meiosis I has been studied, chromosome alignment along the metaphase plate is required to prepare for correct chromosome segregation [14,15]. Each bivalent’s bipolar attachment to the spindle and the balance of forces on each bivalent aids in the alignment and subsequent distribution of chromosomes [16]. The configuration of the sex trivalent, with three kinetochore attachment sites, initially appears to call into question the importance of balancing attachments and forces. Analysis of the movement of sex trivalents in living spermatocytes of the praying mantid *Hierodula membranacea* showed that sex trivalents align with all of the autosomal bivalents in metaphase I and segregate with the bivalents in anaphase I [17]. Nicklas and Arana found, using fixed stained specimens, that sex trivalents in many mantid species do align alongside autosomes in metaphase [6]. Nicklas and Arana further proposed that metaphase alignment may be a regulatory step for successive, successful segregation in anaphase I [6]. Furthermore, studies evaluating the praying mantid *Hierodula membranacea* (Mantodea, Mantidae) revealed that not only does the sex trivalent align with autosomes, but it also segregates simultaneously alongside them during anaphase I, as expected in bivalent configurations [17]. Despite the seemingly unbalanced attachment of the sex trivalent, progression through the meiotic program occurs unhindered, providing support for the evolutionary importance of the metaphase alignment of chromosomes.

The unique configuration of the sex trivalent offers the opportunity to study the meiotic mechanisms regulating the position of chromosomes during metaphase I and the segregation of chromosomes during anaphase I. In this report, we aim to expand previous knowledge of sex trivalents using the previously unstudied praying mantid *Rhombodera megaera*. Here, we show a 2*n* = 27 chromosome number in males of this species. Live-cell imaging reveals that sex trivalents align with autosomes in metaphase I in this species, and that sex trivalent segregation occurs concurrently with autosome segregation. Quantification of immunofluorescence staining suggests that the alignment of the sex trivalent in metaphase is due to balancing microtubule connections on each side of the trivalent, identifying a regulatory step that ensures proper subsequent distribution of chromosomes. This evaluation leads to a deeper understanding of the forces that regulate metaphase alignment, which occurs in many species, and these results may reveal translational implications associated with congenital aneuploidies that arise in mammals.

## 2. Results

### 2.1. Species Identification

**Figure 1:**
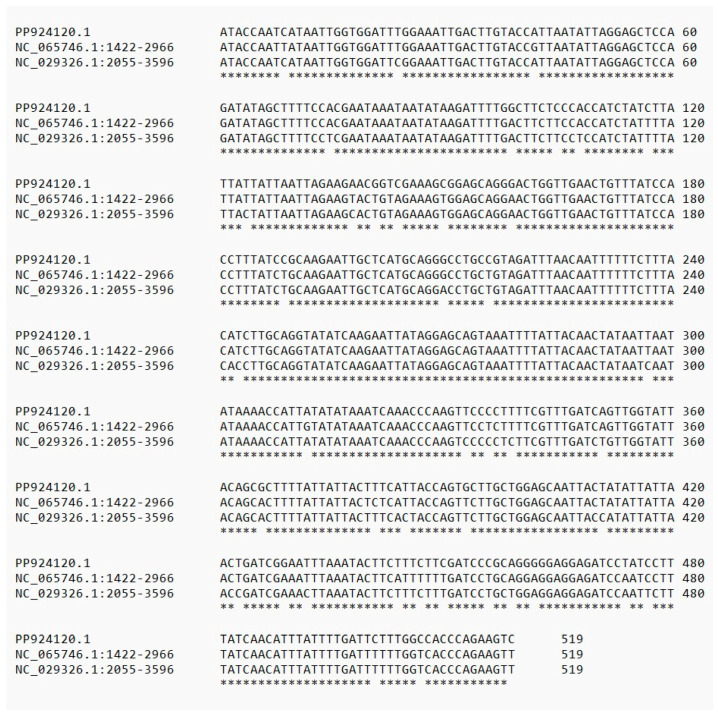
Alignment PP924120.1 (isolate from mantid Arthropod Ambassadors, identified as *Rhombodera megaera*) with nucleotides 1422–2966 of NC_065746.1, the complete mitochondrial genome sequence of *Hierodula zhangi* (95.09% identity), and nucleotides 2055–2596 of NC_029326.1, the complete mitochondrial genome sequence of *Tidanodula formosana* (92.42% identity).

*Rhombodera megaera* were obtained from Arthropod Ambassadors (Salem, OR, USA, https://arthropod-ambassadors.com/ (accessed on 16 September 2024)). Their identity was verified based on Rehn [18] and Svenson [19]. Tissue from the head of an adult was used to generate a DNA barcode sequence. The sequence was entered into Genbank, with accession number PP924120.1. The sequence was then entered in a BLASTN search and an alignment was generated with the two most similar sequences in the BLAST database (Figure 1). The two closest matches were *Hierodula zhangi* (95.09% identical) and *Titanodula formosana* (92.42% identical), two other members of the subfamily Hierodulinae.

### 2.2. Karyotype Analysis

**Figure 2:**
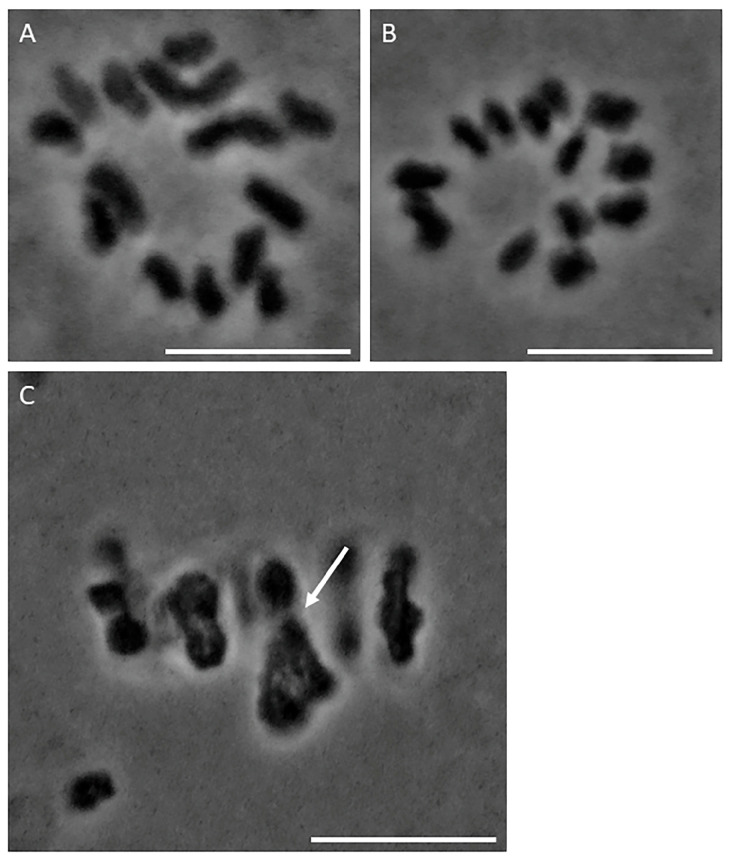
*Rhombodera megaera* spermatocyte chromosome squashes. (**A**) Orcein-stained chromosome squash produced from a female-determining meiosis II prometaphase spermatocyte. The squash shows 14 chromatids. Bar = 5 μm. (**B**) Orcein-stained chromosome squash produced from a male-determining meiosis II spermatocyte. The squash shows 13 chromatids. Bar = 5 μm. (**C**) Orcein-stained chromosome squash produced from a meiosis I spermatocyte. Arrow indicates sex trivalent. Bar = 5 μm.

Spreads and squashes of *Rhombodera megaera* spermatocytes were imaged in meiosis I and meiosis II to determine the chromosome number and the sex determination mechanism. Spread and squashed spermatocytes (>50) generated from three males were used to determine chromosome structure and number. We observed that spermatocytes that develop into female-determining sperm contain 14 chromosomes (Figure 2A), spermatocytes that develop into male-determining sperm contain 13 chromosomes (Figure 2B), and that meiosis I spermatocytes have a sex trivalent (Figure 2C, arrow). These chromosome numbers indicate that *R. megaera* has a diploid chromosome number of 2*n* − 27 in males. These findings are consistent with a previously reported karyotype analysis of the subfamily Hierodulae [4].

### 2.3. Sex Trivalent Alignment

**Figure 3:**
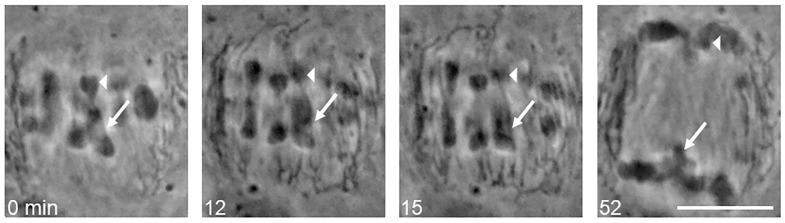
Segregation of chromosomes in meiosis I in the praying mantis *Rhombodera megaera*. The meiosis I spermatocyte has a sex trivalent (0 min arrow and arrowhead), composed of an X_1_ and an X_2_ chromosome (0 min arrow) present on one side of the spindle and a Y chromosome (0 min arrowhead) that is on the opposite side of the spindle. In anaphase, the X_1_ and X_2_ chromosomes (12, 15, and 52 min arrows) segregated toward one spindle pole while the Y chromosome (12, 15, and 52 min arrowheads) segregated toward the opposite spindle pole. In anaphase, the sex chromosomes separated at the same time as the autosomes (12 and 15 min). Scale bar = 5 μm.

Live-cell imaging of *R. megaera* revealed that autosomal bivalents align with the sex trivalent on the metaphase plate during metaphase I (Figure 3, 0 min). Most observed samples (>75), both living and fixed, displayed the orientation of the sex trivalent in the center of the metaphase plate, with autosomal bivalents flanking both sides and alignment at the spindle midline with autosomes. In anaphase I, the sex trivalent and half bivalents segregated nearly simultaneously (Figure 3, 15 and 52 min). The Y chromosome migrated towards one spindle pole while the two X chromosomes were pulled towards the opposing pole until anaphase I completion (Figure 3, 52 min). Sex trivalent behavior was observed in 10 living cells followed from metaphase I through to late anaphase I.

**Figure 4:**
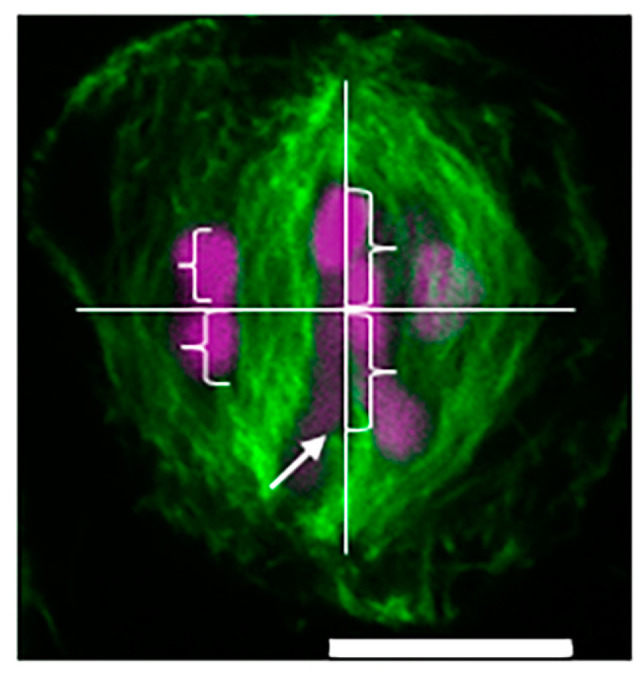
A diagram of an analysis of the position of *Rhombodera megaera* sex trivalents in relation to autosomes in metaphase I. A vertical line was drawn between spindle poles on the spindle axis. A horizontal line was drawn at the midpoint of the axis line between each spindle pole to create a spindle midline. The distance between the position of trivalent kinetochores and autosomal kinetochores from kinetochore attachment to midline (brackets) were measured in pixel length. The ratio of these distances was taken for 12 spermatocytes (*n* = 12 trivalents; *n* = 24 autosomes). Bar = 10 μm.

Analysis of stained metaphase I spermatocytes revealed alignment of the trivalent and autosomes along the metaphase plate. Alignment observations from both live-cell and immunofluorescence imaging were confirmed by quantifying distances between kinetochores and the line perpendicular to the spindle axis at the center of the spindle axis as ratios. Trivalent chromosomes measured a ratio of 0.965 ± 0.031 (calculated from *n* = 12 observations) and autosomal bivalents measured a ratio of 1.027 ± 0.044 (calculated from *n* = 24 observations), indicative of balanced alignment (Figure 4, Table 1).

Quantification of the brightness of stained microtubule bundles revealed that the sex trivalent Y chromosome bound a brighter bundle of stained microtubules than each single X chromosome in the trivalent. Two-tailed *t*-tests and Bonferroni correction, adjusting the threshold of significance to 0.005, were performed on the data, revealing significant differences between the brightness of microtubule bundles associated with either X_1_ or X_2_ and the Y chromosome (*p* = 2.02 × 10^−8^).

## 3. Discussion

**Figure 5:**
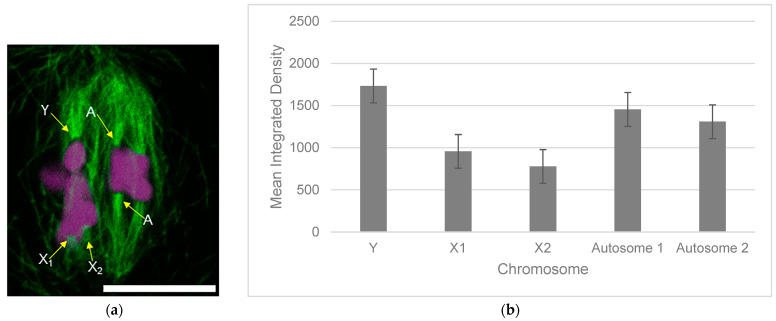
The Y chromosome in a sex trivalent in *Rhombodera megaera* is associated with a brighter-stained microtubule bundle than either X_1_ or X_2_. (**a**) Immunofluorescence imaging of microtubules (green) and DAPI-stained chromosomes (magenta) of a metaphase I spermatocyte. The microtubule bundles associated with the X_1_, X_2_, and Y chromosomes of the sex trivalent as well as an autosome (A) are labeled. Bar = 10 μm. (**b**) Means of measurements of brightness of microtubule bundles associated with the sex trivalent and autosomes. Integrated density measurements were made for microtubule bundles associated with the X_1_, X_2_, and Y chromosomes of the sex trivalent and two autosomes in each cell. Bars represent mean calculations for 12 cells and error bars represent one standard deviation from the mean.

This is the first description of the chromosome number and sex-determining system in the praying mantid *Rhombodera megaera*. Our data show that *R. megaera* has a chromosome number of 2*n* = 27 in males with X_1_X_2_Y (male)/X_1_X_1_X_2_X_2_ (female) sex determination (Figure 2). In primary spermatocytes, thirteen chromosomes align on the metaphase plate in meiosis I. Twelve chromosomes are autosomal bivalents and one is the sex trivalent. The sex trivalent consists of the X_1_ and X_2_ and Y chromosomes. The X_1_ and X_2_ chromosomes aim together toward one spindle pole (Figure 3). The Y chromosome is smaller than either X_1_ or X_2_ and is aimed toward the opposite spindle pole. In metaphase I, all chromosomes, including the sex trivalent, align on the metaphase plate. During anaphase I, the twelve bivalent chromosomes separate and segregate into daughter cells, while the trivalent chromosome complex is dissociated into three parts: X_1_, X_2_, and Y. X_1_ and X_2_ move together to the one pole and the single Y segregates toward another pole, as we observed in living cells (Figure 4 and Figure 5). The chromosome number and sex determination system are consistent with the chromosome numbers, sex determination systems, and patterns of segregation of most of the previously studied related species in the subfamily Hierodulinae [4,17].

Chromosome alignment in metaphase appears to be both conserved and essential for correct cellular functioning following the completion of cell division [20]. The alignment of metaphase chromosomes makes intuitive sense if one thinks of a chromosome with two partner kinetochores as equivalent to a flag tied to the center of a rope in an evenly balanced game of tug of war. These balanced forces occur, in part, because partner kinetochores, and typically all kinetochores in the same cell, associate with equivalent numbers of kinetochore microtubules [21,22]. When a kinetochore is partially destroyed by laser microsurgery, reducing the number of microtubules to which the kinetochore can bind, the chromosome shifts out of the metaphase plate directly away from the spindle pole linked to the kinetochore associated with the smaller number of microtubules [23]. In addition, artificially and newly created multivalent chromosomes with fewer kinetochores associated with one pole than the other will be closer to the pole that has the higher number of kinetochores associated with it [6].

If all of the kinetochores in a cell associate with equivalent numbers of microtubules, and if chromosome alignment is linked to a balance in the number of microtubules bound by paired kinetochores, one would expect that trivalents would not align with bivalents, as was seen in the artificially created multivalents described above [6]. Assuming all kinetochores in a cell are equivalent, the trivalent should shift toward the spindle pole associated with the X_1_ and X_2_ chromosomes and away from the spindle pole associated with the Y chromosome. Previous work, however, showed that in five species of mantids and one species of grasshopper with a sex trivalent, the sex trivalent aligns with all of the autosomal bivalents. These sex trivalents are naturally occurring and ancient; evolution has had time to select for any chromosome behaviors that increase fitness [6]. Sex trivalent alignment has been shown in fixed, stained chromosome squashes, sectioned, stained specimens, and in living cells [6,17].

We hypothesized, based on the previously published work on chromosome alignment and microtubule number at the kinetochore, that the Y kinetochore would associate with approximately twice as many microtubules as either the X_1_ or X_2_ kinetochores. While we did not have access to a method (electron microscopy) that would allow us to precisely count the number of microtubules occupying each kinetochore for this study, we were able to immunostain metaphase I spermatocytes and measure the brightness of microtubule bundles imaged with a fluorescence microscope (Figure 5). This method is not precisely quantitative but has been previously used for broad comparisons of microtubule occupation at kinetochores [24]. Our measurements revealed that the Y kinetochores of sex trivalents are associated with a significantly brighter bundle of microtubules than either the X_1_ or X_2_ kinetochore, suggesting that the balanced position of the chromosome can be explained, at least in part, by differences in the number of microtubules occupying the kinetochores of each kinetochore of the sex trivalent.

Differences in the number of microtubule binding sites could be built into the centromeres of X_1_, X_2_, and Y, in a manner similar to what is observed in mice that exhibit meiotic drive and preferential segregation of some centromeres to the spindle pole in the egg in meiosis I. In this mouse system, the homologue that segregates to the egg and not the polar body has a stronger centromere [25]. The stronger centromere contains more functional satellite DNA repeats and more CENP-A-containing nucleosomes [26]. In female meiosis, because the spindles have asymmetric post-translational modifications of tubulin, this leads to the stronger centromeres being more likely to detach from microtubules emanating from the polar-body-oriented spindle pole and thus being more likely to segregate into the egg [27]. In the praying mantis, centromeres of X_1_ and X_2_ could have fewer satellite repeats than the Y centromere, leading to fewer CENP-A-containing nucleosomes and a smaller kinetochore that presumably has fewer microtubule-binding sites. On a spindle that is likely to be symmetric for post-translational modifications of tubulin, differences in centromere strength between the X chromosomes and the Y chromosome could simply lead to a balance in the strength of attachment, such that X_1_ and X_2_ opposing Y is the only attachment that is under strong tension and stable. Alternatively, the X centromeres and the Y centromeres could have some different epigenetic adaptations, or some X-specific kinetochore protein modifications only associated with meiosis. These adaptations/modifications could reduce the number of microtubules bound on the X_1_ and X_2_ kinetochores only in meiosis, and then have very similar kinetochore structure and microtubule-binding capabilities to one another and all autosomal kinetochores in mitosis. Further analysis will be required to determine the underlying mechanisms for the likely variation in microtubule number at each kinetochore of the sex trivalent.

In conclusion, we showed that a naturally occurring, ancient sex trivalent aligns on the metaphase plate, providing support for the evolutionary importance of chromosome alignment in metaphase. This alignment is explained, at least in part, by differences in microtubule occupation of the X_1_, X_2_, and Y kinetochores.

## 4. Materials and Methods

### 4.1. Collection and Identification

*Rhombodera megaera* adults were obtained from Arthropod Ambassadors (Salem, OR, USA, https://arthropod-ambassadors.com/ (accessed on 16 September 2024)). Adult individuals were mated and the resulting colony (>150 individuals from 4 mated females) was raised to the subadult stage. Subadult males from the colony were dissected and testes were removed for both living-cell and fixed-cell experiments. Species identification was based on Rehn [18] and Svenson [19].

### 4.2. DNA Barcoding

To collect additional identifying data about the species, DNA barcoding was completed. DNA was extracted from the head of a mantid using the Zymo Quick-DNA Tissue/Insect Miniprep Kit (Zymo Research, Irvine, CA, USA) according to manufacturer’s protocol, with the additional step of bead beating (2 min/1500 rpm). PCR was performed using a Carolina Using DNA Barcodes to Identify and Classify Living Things Kit (Carolina Biological Supply, Burlington, NC, USA)) according to manufacturer’s protocol. PCR products were electrophoresed on a 1% agarose/1× TBE gel to confirm the size and quantity. PCR products were purified using the Zymo DNA Clean and Concentrator Kit (Zymo Research, Irvine, CA, USA according to the manufacturer’s protocol. The PCR product was ligated into a pMiniT 2.0 Vector using a NEB PCR Cloning Kit (New England Biolabs, Ipswich, MA, USA) according to the manufacturer’s instructions. Resulting colonies were grown in LB + 100 μg/mL ampicillin overnight in a shaking incubator at 37 °C. Plasmids were isolated using QIAgen Spin Miniprep (Qiagen, Germantown, MD, USA according to the manufacturer’s protocol. DNA was sequenced by Eurofins Genomics (Eurofins Genomics, Louisville, KY, USA) using NEB forward and reverse primers.

### 4.3. Orcein Staining of Chromosome Spreads

*Rhombodera megaera* testes were fixed in 3:1 ratio of ethanol/acetic acid. After 10 min, the testes were macerated on a microscope slide submerged with 60% acetic acid. Cells were spread onto a slide and placed onto a slide dryer at 45 °C until dried. Chromosomes were stained for 10 min in 3% Giemsa stain. Following three five-minute rinses in water, slides were mounted using glycerol as a mounting medium and observed using a Nikon Eclipse TS 100 (Nikon Instruments, Tokyo, Japan) inverted phase-contrast microscope equipped with a 100X, 1.25 NA phase-contrast, oil immersion objective and a View4K HD camera (Microscope Central, Feasterville, PA, USA), and InFocus software version ×64 (Microscope Central) [28].

### 4.4. Orcein Staining of Chromosome Squashes

*Rhombodera megaera* testes were removed and placed in a 3:1 ethanol/acetic acid solution for 10 min. After a 30 s rinse in distilled water, testes were transferred into aceto-orcein stain for 5 min and then onto a slide containing 45% acetic acid for testis distribution using laboratory forceps. Testis follicles were left in acetic acid for 30 s. A coverslip was placed over the top and was squashed using absorbent paper for assistance. Slides were sealed with nail polish and observed using a Nikon Eclipse TS100 microscope (Nikon Instruments, Tokyo, Japan) equipped with a 100X, 1.25 NA phase-contrast, oil immersion objective and a View4K HD camera (Microscope Central), and InFocus software version ×64 (Microscope Central). Adobe Photoshop CC 2023 was used to take advantage of the full range of pixel values and to crop and rotate images [17].

### 4.5. Preparation of Living Cells

*Rhombodera megaera* testes were removed and submerged into a culture chamber containing a layer of Kel-F Oil #10 (Ohio Valley Specialty Company, Marietta, OH, USA). Spermatocytes were spread on a coverslip under a thin layer of oil [29]. Live-cell imaging of the spermatocytes during meiosis I was completed using a Nikon Eclipse TS 100 inverted phase-contrast microscope (Nikon Instruments, Tokyo, Japan) equipped with a 100X, 1.25 NA phase-contrast, oil immersion objective and outfitted with a View4K HD camera (Microscope Central), and InFocus software version ×64 (Microscope Central). Adobe Photoshop CC 2023 was used to take advantage of the full range of pixel values and to crop and rotate images.

### 4.6. Immunofluorescence

The testes of *Rhombodera megaera* were removed from the abdomen and contents were spread under a thin layer of Kel-F Oil #10 to produce a single-cell thin layer of living spermatocytes. Fixation was as described by Nicklas et al. [30]. Cells were stained as described in King and Nicklas [24] and mounted onto a glass slide with Vectashield (Vector Laboratories, Newark, CA, USA) [28].

### 4.7. Quantification of Brightness of Microtubule Bundles

A total of 75 cells from metaphase I were imaged. Imaging of stained cells was performed using a 100X, 1.4 NA oil immersion objective on a Leica SP5 Spectral Systems confocal microscope. Z-stacks were collected with 0.25 μm intervals. Images were analyzed using Adobe Photoshop CC 2023. Cells in which all three kinetochore ends of the trivalent were in focus in the same field of view were selected for further analysis. Photos were rotated so that each microtubule bundle associated with each chromosome of the sex trivalent was vertically centered and subjected to integrated density analysis. A standardized pixel box with the dimensions of 2X5 was created for all measurements and placed 3 pixels above the tip of each trivalent chromosome to capture fluorescence intensity. Three measurements of background intensity were taken for each cell and average background intensity was subtracted from measurements of the density of microtubule bundles. Integrated density for Y, X_1_, X_2_, and autosomal microtubule bundles were averaged with standard deviations for 12 cells.

### 4.8. Analysis of Chromosome Position

Chromosome position was calculated in both fixed, stained cells and in living cells. In living cells, positions of spindle poles were estimated by identifying the vertex of the cleared areas within cells. In fixed, stained cells, the point of convergence of the paths of the kinetochore microtubules was considered the position of the spindle pole. In both cell types, a line was drawn approximating the horizontal and vertical axes of the spindle pole using Adobe Photoshop CC 2023. The distances between the chromosome edges (estimated kinetochore attachment sites) and associated spindle midline were measured for the chromosomes of the trivalent and two additional autosomes in the same plane of the cell. The ratio of these distances was calculated [17].

### 4.9. Statistical Analysis

A statistical analysis was performed using a two-tailed *t*-test, assuming equal variances, to compare distance ratios between kinetochores and the midline in sex trivalents and autosomes of *R. megaera*. The threshold of significance was set at 0.05.

A statistical analysis was performed using a one-way ANOVA to compare average integrated densities of sex trivalent microtubule bundles to autosomal. Post hoc two-tailed *t*-tests were performed along with a Bonferroni correction to account for multiple comparisons. The threshold of significance after correction was 0.005. The data for both analyses did not present with any outliers.

## Figures and Tables

**Table 1:** *Rhombodera megaera* sex trivalents and autosomes align together along the metaphase plate. Distances between kinetochores and the spindle midline for both trivalents and autosomes were used to calculate approximate ratios. A two-tailed *t*-test assuming equal variances was performed to compare distance ratios between kinetochores and the midline in sex trivalents and autosomes. The test revealed that there was no statistically significant difference in distance ratios (*t*-Stat = −0.884, df = 34, *p*-value = 0.383). *n* = 12 trivalents and *n* = 24 autosomes were measured in 12 cells.

Distance between X Kinetochores and Midline/Distance between Y Kinetochore and Midline	Distance between Autosomal Kinetochore and Midline/Distance between Partner Autosomal Kinetochore and Midline
0.965 ± 0.031 (*n* = 12 trivalents)	1.027 ± 0.044 (*n* = 24 autosomes in 12 cells)

## Data Availability

The data presented in this manuscript are available upon request from the corresponding author.
